# Impact of Insecticides at Sublethal Concentrations on the Enzyme Activities in Adult *Musca domestica* L.

**DOI:** 10.3390/toxics11010047

**Published:** 2023-01-01

**Authors:** Anna Kinareikina, Elena Silivanova

**Affiliations:** All-Russian Scientific Research Institute of Veterinary Entomology and Arachnology—Branch of Federal State Institution Federal Research Centre Tyumen Scientific Centre of Siberian Branch of the Russian Academy of Sciences (ASRIVEA—Branch of Tyumen Scientific Centre SB RAS), Institutskaya st. 2, 625041 Tyumen, Russia; Tel./Fax: +7-3452-258558

**Keywords:** housefly, insecticides, chlorfenapyr, fipronil, acetylcholinesterase, carboxylesterase, glutathione-S-transferase, monooxygenase sublethal effect

## Abstract

Nowadays, the use of pesticides is, as before, the most common way to control arthropod plant pests and the ectoparasites of animals. The sublethal effects of pesticides on insects can appear at different levels, from genetics to populations, and the study of these effects is important for a better understanding of the environmental and evolutionary patterns of pesticidal resistance. The current study aimed to assess the sublethal effects of chlorfenapyr and fipronil on the activities of detoxifying enzymes (carboxylesterase—CarE, acetylcholinesterase—AChE, glutathione-S-transferase—GST, and cytochrome P450 monooxygenase—P450) in adults *Musca domestica* L. The insects were exposure to insecticides by a no-choice feeding test and the enzyme activities and the AChE kinetic parameters were examined in female and male specimens at 24 h after their exposure. According to Tukey’s test, the CarE activity was statistically significantly decreased by 29.63% in the females of *M. domestica* after an exposure to chlorfenapyr at a concentration of 0.015% when compared to the controls (*p* ≤ 0.05). An exposure to the sublethal concentration of fipronil (0.001%) was followed by a slightly decrease in the specific activity (33.20%, *p* ≤ 0.05) and the main kinetic parameters (Vmax, Km) of AChE in females in comparison with the control values. The GST and P450 activities had not significantly changed in *M. domestica* males and females 24 h after their exposure to chlorfenapyr and fipronil at sublethal concentrations. The results suggest that the males and females of *M. domestica* displayed biochemically different responses to fipronil, that is a neurotoxin, and chlorfenapyr, that is a decoupler of oxidative phosphorylation. Further research needs to be addressed to the molecular mechanisms underlying the peculiarities of the insect enzyme responses to different insecticides.

## 1. Introduction

Nowadays, the use of pesticides remains the most common way to control arthropod plant pests and ectoparasites [1,2]. The use of pesticides in agriculture has increased over the past few decades with the continuous growth of the global food production [3]. According to the Food and Agricultural Organization, in the year of 2019, insecticides were the third most commonly used pesticide [4]. The loss of chemical substances during the emission of pesticides into the environment can range from 2% to 25% [3] and can lead to significant environmental pollution risks and potentially hazardous effects on human and animal health [2]. Under the influence of abiotic and biotic factors, the pesticide concentrations in environmental objects changes over time [5,6]. Field insect populations are under the acute and chronic influence of lethal and sublethal pesticide concentrations. It can lead to various consequences: insect death, changes in their life cycle, fertility, behavior, physiological, biochemical, molecular, and genetic parameters of both the parent generation and their offspring [7,8,9]. The study of the chronic and sublethal effects of an insecticidal exposure and their species-specific characteristics is important for a deeper understanding of the environmental and evolutionary patterns of pesticidal resistance. These patterns can shape the modern approaches to the pest and mite population control, prevention, and elimination of the resistance [8,10].

Plenty of the currently available insecticides are neurotoxins which affect certain parts of the nervous system. For example, fipronil of the phenylpyrazole class has a wide contact-intestinal, systemic spectrum of action. It acts as a non-competitive γ-aminobutyric acid receptor antagonist and its binding to the GABA receptors results in the blockage of the chloro-ion channels of nerve cells [11]. The mechanism of action of other insecticidal compounds is not associated with the impact on the nervous system. For example, chlorfenapyr, a pro-insecticide, belongs to the metabolic process modulators. The Insecticide Resistance Action Committee (IRAC) classifieds chlorfenapyr as a decoupler of oxidative phosphorylation due to the ability of its metabolites to block the ATP synthesis in the cells of insects [12]. Chlorfenapyr is effectively used as a non-repellent insecticide against various plant pests and synanthropic insects (cockroaches, bedbugs, termites, ants, mosquitoes, etc.) in America, Europe, the Asia-Pacific region, Africa, and the Middle East [13,14]. A previous study showed the synergistic interaction pattern in the fipronil/chlorfenapyr (1:4) mixture against the adults of houseflies (*Musca domestica* L.) [15]. Thus, chlorfenapyr and fipronil (in toxic bait formulations) may serve as an alternative to common insecticides against the adults of *M. domestica*.

The hydrolysis of insecticides by esterase is an important biochemical mechanism for the development of insecticide resistance that is common to several classes of chemical compounds [16,17,18]. Insect carboxylesterase (EC 3.1.1.1, CarE) performs the hydrolysis of carboxyl ester bonds and is involved both in the detoxification of exogenous compounds and in the metabolism of compounds of a physiological importance [18,19,20]. The participation of CarEs in the metabolism of organophosphorus compounds (OPCs), pyrethroids, and the development of a resistance to them has been demonstrated [21]. Carboxylesterases can rapidly bind the OPC molecules and slowly hydrolyze the resulting phosphoester bond [22]. In *Aedes aegypti* and *Anopheles gambiae* mosquitoes, carboxylesterase metabolize pyrethroids form phenoxybenzyl alcohol and phenoxybenzaldehyde, which can be further converted to phenoxybenzoic acid under the influence of P450 cytochromes. In “in vitro” experiments, the ability of different *Musca domestica* L. (Diptera:Muscidae) carboxylesterase isoforms to hydrolyze permethrin, but not its metabolites, was demonstrated [20]. The possibility of the “in vitro” metabolism of β-cypermethrin and fenvalerate under the influence of carboxylesterase of the cotton bollworm *Helicoverpa armigera* (Lepidoptera: Noctuidae) was demonstrated in the work of Li et al. [23]. Acetylcholinesterase (EC 3.1.1.7, AChE) is a serine esterase of the α-, β-hydrolase family. Its main role is to regulate the acetylcholine levels in cholinergic synapses and thus to partake in the nerve impulse transmission [24]. AChE in insects is a specific molecular target of OPCs and carbamates. The resistance development to these compounds is often realized through the mechanism of decreasing the enzyme’s sensitivity to them [24,25]. An assumption was made that solubilized AChE isoforms in insects can be involved in the sequestration of xenobiotics, including insecticides [26,27]. In addition to esterases, cytochrome P450 monooxygenase (EC 1.14.14.1, P450) and glutathione-S-transferase (EC 2.5.1.18, GST) contribute to protecting insects against insecticides [16]. For instance, an increased P450 monooxygenase activity was observed in insects resistant to thiamethoxam [28], pyrethroids [29], and spinosad [30]. The GST activities in insecticide-resistance insects are reported as being either no different [31,32] or increased compared to that in insecticide-susceptible insects [31,33].

Model insect species, such as the housefly *Musca domestica* L., are often used to test insecticides and study the insecticide resistance [34]. Numerous publications indicate the evolution of insecticide resistance in field populations of the housefly worldwide [35]. Under controlled laboratory conditions, *M. domestica* is capable of developing a resistance rather quickly (within 5–7 generations) in response to an exposure to certain insecticides [28,36]. The current study was carried out to assess the sublethal effects of chlorfenapyr and fipronil on the detoxification enzyme activities (CarE, AChE, GST, and P450 monooxygenase) in *M. domestica* adults. The study also examines the AChE kinetic parameters in female and male houseflies after their exposure to sublethal concentrations of chlorfenapyr and fipronil. We observed slight changes in the activities and main kinetic parameters of AChE as a sublethal effect of fipronil, that is a neurotoxin, but not of chlorfenapyr, that is a decoupler of oxidative phosphorylation. This study confirmed the differences in the enzymatic response to a sublethal exposure between *M. domestica* females and males.

## 2. Materials and Methods

### 2.1. Chemicals

The following chemical compounds and reagents were used: EDTA (≥99.0%, BioUltra), PTU (N-Phenylthiourea, ≥98.0%), PMSF (Phenylmethylsulfonyl fluoride, >98.5%), DTE (1,4-Dithioerythritol, ≥99.0%), Triton X-100 (t-Octylphenoxypolyoxyethethanol, ≥100.0%), DTNB (5,5′-Dithiobis(2-nitrobenzoic acid), ≥98.0%), Acetylthiocholine iodide (≥98.0%), GSH (Glutathione reduced, ≥98.0%), 1-Chloro-2,4-dinitrobenzene (97%), 3,3′,5,5′-tetramethyl-benzidine dihydrochloride (TMBZ), and Cytochrome C were obtained from Sigma-Aldrich (Germany); Folin–Ciocalteu’s Reagent (PanReac, AppliChem, Italy); BSA (bovine serum albumin) (ZAO Diakon-DC, Russia); mono- and disubstituted sodium and potassium phosphates, sulfurous copper, sodium carbonate (OOO AO REACHIM, Russia).

### 2.2. Insects and Insecticide Treatments

The objects of the study were laboratory adult specimens, 3–5 days old, of housefly *Musca domestica* L. (average weight of a female was 13.43 ± 4.24 mg, a male was 8.64 ± 2.32 mg). The insects were kept in boxes with a constant temperature of 27 ± 1 ℃ and a relative humidity of 50 ± 5%. The insects were exposed to insecticides (chlorfenapyr or fipronil) by a no-choice feeding test [37,38]. Briefly, the sugar (0.1 g) was placed in glass cups and was treated with 30 µL of the acetone solutions of insecticides, namely chlorfenapyr at concentrations of 0.015% and 0.025%, and fipronil at concentrations of 0.0005% and 0.001%. After the acetone evaporated, ten flies were placed into each cup. In the control tests, the sugar was treated with pure acetone. The cups were sealed with mesh pistons from the top and supplied with water drinkers. The mortality of the flies was recorded after 24 h, and the surviving flies were kept at −80 °C. The experiments with each concentration were repeated at least three times.

### 2.3. Assay of Enzyme Activities

Homogenates were prepared from each specimen of *M. domestica* manually at low temperatures with the addition of 0.1 M of phosphate buffer pH = 7.6, containing 1 mM of EDTA, 1 mM of PTU, 1 mM of PMSF, 1 mM of DTE, and 20% Triton X-100. The supernatant obtained after centrifugation (2 min, 12,500 rpm) was used to determine the enzyme activities and protein concentration; the supernatant before centrifugation was used to determine the monooxygenase activities. The protein content was determined by the Lowry protein assay, using bovine serum albumin solutions to construct a calibration curve. The determination of the enzyme activity was performed on 96-well microplates (MiniMed, Suponevo, Russia) on a Multiskan FC microplate photometer (Thermo Fisher Scientific Inc., Waltham, MA, USA).

The CarE activity was assessed towards p-nitrophenylacetate at 405 nm in the kinetic mode for 5 min at 30 °C [39]. The reaction mixture contained 50 µL of homogenate and 200 µL of 50 mM sodium phosphate buffer (pH = 7.4) with 1 mM of p-nitrophenylacetate. To account for the non-enzymatic hydrolysis of the substrate, 10 µL of sodium phosphate buffer (pH = 7.4) was added to the reaction mixture instead of a homogenate.

The AChE activity was assessed according to the Ellman’s method with minor modifications [40]. To assess the specific activity of the enzyme, the reaction mixture contained 10 µL of homogenate, 90 µL of 50 mM potassium phosphate buffer (pH = 7.0), and 100 µL of Ellman’s reagent (2 mM of acetylthiocholine iodide and 0.23 mM of DTNB mixed just before the measurement). To account for the non-enzymatic hydrolysis of acetylthiocholine, 10 µL of potassium phosphate buffer (pH = 7.0) was added to the reaction mixture instead of a homogenate. The substrate content in the reaction mixture when determining the AChE activity to analyze the kinetic parameters (Michaelis constant, Km and maximal velocity, Vmax) was 0.0625 mM, 0.125 mM, 0.25 mM, 0.5 mM, 1 mM, and 2 mM. To determine the specific activity of AChE, the optical density was measured at 405 nm in the kinetic mode for 30 min at 30 °C. The absorbance in the case of the determination of the kinetic parameters was measured at 405 nm in the kinetic mode for 5 min with 15 s intervals at 30 °C. The AChE activity was represented as ΔOD/min/mg of the protein (change in the optical density per minute per mg of protein).

The GST activity was assessed towards 1-chloro-2,4-dinitrobenzene (CDNB) at 340 nm in the kinetic mode for 20 min at 25 °C [39]. The reaction mixture contained 15 µL of homogenate and 195 µL of 100 mM potassium phosphate buffer (pH = 6.5) with 9 mM of GSH in 1mM of CDNB. To account for the non-enzymatic conjugation, 15 µL of water was added to the reaction mixture instead of a homogenate.

The functional activity of the cytochrome P450 monooxygenases was assessed by the total content of the heme at 620 nm in the end point mode [39]. The reaction mixture contained 20 µL of homogenate, 60 µL of 90 mM potassium phosphate buffer (pH = 7.2), 200 µL of working solution 0.2% TMBZ with 250 mM of sodium acetate buffer (pH = 5.0), and 25 µL of 3% hydrogen peroxide. Cytochrome C solutions were used to construct a calibration curve. The P450 monooxygenase activity was represented as the µg of the cytochrome C/mg of the protein.

### 2.4. Data Analysis.

The kinetic parameters were determined by non-linear regression using Excel Solver software [41,42]. The statistical analysis of the enzyme activity results was performed by a one-way ANOVA test and Tukey’s test for multiple comparisons using the Statistica 13.3 software package (StatSoft, Moscow, Russia). The significance level of *p* < 0.05 was used to consider the identified differences as statistically significant.

## 3. Results

As shown in Table 1, chlorfenapyr at the concentration of 0.015% caused the 20% and 42% mortality of females and males of *M. domestica*, respectively, at 24 h after their insecticide exposure. An exposure to chlorfenapyr at the concentration of 0.025% led to the 40% and 100% mortality of the females and males of the insects, respectively. The mortality of the houseflies was 23% and 45% when the females and males of *M. domestica*, respectively, were exposed to fipronil at the concentration of 0.0005%. The mortality of females and males was 72% and 100%, respectively, after their exposure to fipronil at the concentration of 0.001%.

**Figure 1:**
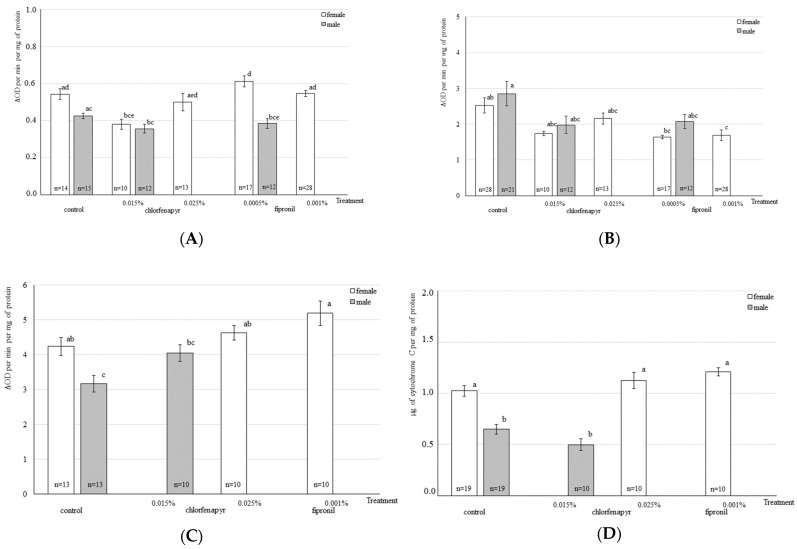
The enzyme activities in adults Musca domestica after exposure to insecticides at sublethal concentrations. Bars show the means with standard error (SE). Bars with the same letters are not significantly different according to Tukey’s post hoc HSD comparisons at *p* < 0.05: (**A**) carboxylesterase; (**B**) acetylcholinesterase; (**C**) glutathione-S-transferase; (**D**) cytochrome P450 monooxygenase.

According to Tukey’s test, the CarE activity was statistically significantly decreased by 29.63% in the females of *M. domestica* after an exposure to chlorfenapyr at a concentration of 0.015% when compared to the controls (*p* ≤ 0.05) (Figure 1A). After an exposure to a higher concentration of insecticide (0.025%), no statistically significant difference in the carboxylesterase activity was observed in the females of the experimental and control groups. No statistically significant changes in the CarE activity were observed in the females after an exposure to fipronil as well as in male *M. domestica* after an exposure to both chlorfenapyr and fipronil when compared to the controls (Figure 1A).

The specific activity of AChE and its kinetic parameters in the female *M. domestica* of the control group and the group exposed to chlorfenapyr were not significantly different (Figure 1B). In females, after an exposure to fipronil at the concentration of 0.001%, the specific activity of the enzyme was statistically significant lower by 33.20% when compared with the control level (*p* ≤ 0.05) (Figure 1B). There was also a decrease in the Km value by 43.59% (*p* = 0.66) and the Vmax value by 45.29% (*p* = 0.64) in females exposed to fipronil at a concentration of 0.001% as compared to the control females (Table 2). No statistically significant changes in the specific acetylcholinesterase activity and Km and Vmax parameters were found in males after an exposure to both chlorfenapyr and fipronil (Table 2).

The GST and P450 monooxygenase activities in male *M. domestica* of the control group were statistically significantly lower by 25.30% and 36.6%, respectively, when compared with the enzyme activities in females of the same group (Figure 1C,D). No statistically significant changes in the GST and P450 monooxygenase activities were observed in males and females after an exposure to both chlorfenapyr and fipronil (Figure 1C,D).

## 4. Discussion

Insects in the natural environment are exposed to lethal and sublethal concentrations of insecticides, in response to which they are able to develop a resistance. The monitoring of insects for the insecticide resistance includes an assessment of the enzyme activity in the insects in addition to evaluating of susceptibility to insecticides by toxicological methods [43]. Insect esterases (CarE, AChE), glutathione-S-transferases, and cytochrome P450 monooxygenases are the main groups of the detoxification enzymes, which contribute to the development of a resistance, and therefore serve as biomarkers for the monitoring of the environmental quality [44,45] and insecticide resistance monitoring [46]. The current study was designed to assess the responses of CarE, AChE, GST, and P450 in *M. domestica* adults after an acute exposure of chlorfenapyr and fipronil at sublethal concentrations.

Previous studies demonstrated an activity increase and qualitative changes in the detoxification enzymes in the insects resistant to OPCs, carbamates [47], pyrethroids [29,46,48,49,50], and neonicotinoids [28,51]. Moreover, researchers reported different enzymatic responses to different insecticides at sublethal concentrations. In our study, the GST and P450 activities were not statistically significantly changed in male and female *M. domestica* at 24 h after their exposure to chlorfenapyr and fipronil at sublethal concentrations. This is comparable to the results by other researchers. Farooq and Freed (2018) reported that a treatment with fipronil at LC10, LC30, and LC50 concentrations caused no changes in the GST activities in adult *M. domestica* [52]. In the study by Zhao et al. (2018), the GST activity in the maggots of *Bradysia odoriphaga* (Diptera: Sciaridae) treated with chlorfenapyr at LC20 and LC50 concentrations did not significantly differ from the control [53]. The GST and P450 activities were shown to be decreased in the fourth instar larvae of *Chilo suppressalis* after being topically treated by fipronil at an LD80 dosage [54].

According to the results obtained, an exposure to chlorfenapyr at a concentration of 0.015% and fipronil at a concentration of 0.001% led to a decrease in the CarE activity and the AChE activity, respectively, in female *M. domestica*. Generally, insecticides, when applied at sublethal concentrations, can cause the induction of the detoxifying enzymes in insects. For instance, Siddiqui et al. (2022) examined the mechanism of the sublethal effects of beta-cypermethrin and fipronil on the red imported fire ants *Solenopsis invicta* Buren (Formicidae: Hymenoptera) and showed that the activity of AChE and CarE increased with an increase in the insecticide concentration [46]. In a study by Farooq and Freed, the AChE activities was elevated in adult *M. domestica* treated with biphentrine, acetamiprid, and fipronil at sublethal doses in comparison to the control specimens [52]. Zhao et al. reported an increase in the CarE activity in the maggots of *B. odoriphaga* after an exposure to chlorfenapyr at sublethal concentrations [53]. Other researchers showed no sublethal effects of the insecticides on the esterase activities. Dewer et al. (2016) emphasized that there was no impact of chlorpyriphos and methomyl on the AChE activity in the maggots of *Spodoptera littoralis* Boisduval (Lepidoptera: Noctuidae) when these insecticides were applied at sublethal doses [55]. The results of the study by Li et al. (2022) showed that the activities of AChE and CarE were not affected by the chlorfenapyr at the sublethal concentration applied on the nymphs of the papaya mealybug, *Paracoccus marginatus* Williams and Granara de Willink (Hemiptera:Pseudococcidae) [9]. In a study by Carvalho et al. (2013), fipronil did not modulate the AChE and one of the CarE isoforms (CarE-2) and, at the same time, increased the CarE-2 activity and decreased the CarE-3 activity in the honeybee *Apis mellifera* L. (Apidae:Hymenoptera) [44]. In an investigation by Roat et al., fipronil at a sublethal dose modulated the activity of CarE in honeybees and did not affect the activity of AChE [56]. These results agree to some extent with the data obtained by Feng et al. (2018); according to which, permethryn applied at sublethal doses caused the induction of eight CarE genes and does not affect the expression of the AChE gene in adult *M. domestica* [57]. It is worth keeping in mind that the induction of the detoxifying enzyme genes might be tissue-specific and depending on the insecticide doses and the time after exposure [57].

The qualitative changes in the enzyme molecule in insects as a response to an insecticide exposure can also be manifested through a shifting in the enzyme activity and affinity toward specific substrates, which, in their turn, can be observed through the kinetic parameters. We noted that for the control insects, the Km value of AChE was 2.7 times more in the females when compared with that in the males (*p* < 0.05). A similar proportion was observed for the specimens exposed to chlorfenapyr at sublethal concentrations, namely, the Km value was 3.4 times more in females that that in the males (*p* < 0.05). In contrast, the Km values of AChE in *M. domestica* after an exposure to fipronil did not differ between the males and females. This might be a consequence of a decrease in the Km value by 43.59% (*p* = 0.66) in the females exposed to fipronil as compared to the control. Additionally, we noted a decrease in the Vmax value by 45.29% (*p* = 0.64) in the females after an exposure to fipronil. Previously, the kinetic parameters of AChE were usually determined for the insects resistant to OPs and carbamates for the development of a resistance based on a mechanism of the decreased sensitivity of the molecular target (i.e., AChE) to insecticides. For instance, Shi et al. (2002) reported changes in the affinity and rate of hydrolysis of three substrates by the acetylcholinesterase of the propoxur-resistant specimens of the *M. domestica* strain: the Km and Vmax values of AChE for acetylthiocholine (ATC) in the adults of the resistant strain were higher than in the specimens of the insecticide susceptible strain [47]. The above-mentioned study has concluded that in the propoxur-resistant strain, there was a decrease in the affinity of the enzyme to the insecticides and substrate, as well as a decrease in the catalytic efficiency of AChE against the specific substrate (ATC). Similar results were obtained in other studies where authors investigated AChE in OPs- and carbamate-resistant insects [58,59,60]. A simultaneous decrease in the Vmax and Km are usually effects of an uncompetitive inhibitor due to the changes in the enzyme-substrate complex equilibrium [61].

## 5. Conclusions

An exposure to chlorfenapyr at sublethal concentrations led to a decrease in the carboxylesterase activity only in female *M. domestica*, but there were no statistically significant changes in the specific activity of acetylcholinesterase in the adults of both sexes when compared with the control. An exposure to chlorfenapyr did not affect the GST and P450 activities and the kinetic parameters of acetylcholinesterase in adult *M. domestica*. No statistically significant changes in the CarE, GST, and P450 activities in the adult (both female and male) *M. domestica* compared with the corresponding control specimens were recorded after an acute exposure to sublethal concentrations of fipronil. An exposure to the sublethal concentration of fipronil (0.001%) was followed by a 33.20% decrease in the specific acetylcholinesterase activity in females in comparison with the control values (*p* ≤ 0.05). A fipronil exposure was also followed by a decrease in the Vmax (by 45.29%, *p* = 0.64) and Km (by 43.59%, *p* = 0.66) of acetylcholinesterase in females, but not in males, when compared with the corresponding control specimens. Taken together, the results obtained and the published data allow us to suggest that insect males and females display biochemically different responses to an insecticidal exposure. Further research must to address to molecular mechanisms underlying the peculiarities of the insect enzyme responses to different insecticides.

## Figures and Tables

**Table 1:** Mortality of adults *Musca domestica* at 24 h after exposure to insecticides.

Treatment		Number of Insects			
		Females		Males	
		Total	Dead	Total	Dead
Control		30	0 (0%)	30	0 (0%)
Chlorfenapyr	0.015%	34	7 (20.0%)	49	21 (42.9%)
	0.025%	32	13 (40.6%)	29	29 (100%)
Fipronil	0.0005%	30	7 (23.3%)	40	18 (45.0%)
	0.001%	60	43 (71.7%)	31	31 (100%)

**Table 2:** Kinetic parameters of acetylcholinesterase in adults of *M. domestica* (M ± SD) *.

Treatment		n	Vmax, ΔOD/min/mg of Protein	Km, mM of ATC
Control		13♂	2.48 *±* 0.67	0.43 *±* 0.15 ^a^
		18♀	3.93 *±* 2.22	1.17 *±* 0.88 ^b^
Chlorfenapyr	0.015%	12♂	2.51 *±* 0.50	0.40 *±* 0.19 ^a^
	0.025%	10♀	4.74 *±* 2.44	1.36 *±* 1.06 ^b^
Fipronil	0.0005%	12♂	3.87 *±* 2.76	0.76 *±* 0.61 ^ab^
	0.001%	25♀	2.86 *±* 2.21	0.83 *±* 0.49 ^ab^

* Vmax—the maximal velocity; Km—the Michaelis constant; OD—optical density; ATC—acetylthiocholine iodide; means within the column followed by the same letters are not significantly different according to Tukey’s post hoc HSD comparisons at *p* < 0.05.

## Data Availability

Not applicable.

## References

[1] Lykogianni M., Bempelou E., Karamaouna F., Aliferis K.A. (2021). Do pesticides promote or hinder sustainability in agriculture? The challenge of sustainable use of pesticides in modern agriculture. Sci. Total Environ..

[2] Zikankuba V.L., Mwanyika G., Ntwenya J.E., James A. (2019). Pesticide regulations and their malpractice implications on food and environment safety. Cogent Food Agric..

[3] Casu V., Tardelli F., De Marchi L., Monni G., Cuccaro A., Oliva M., Freitas R., Pretti C. (2019). Soluble esterases as biomarkers of neurotoxic compounds in the widespread serpulid *Ficopomatus enigmaticus* (Fauvel, 1923). J. Environ. Sci. Health B.

[4] Indira Devi P., Manjula M., Bhavani R.V. (2022). Agrochemicals, Environment, and Human Health. Annu. Rev. Environ. Resour..

[5] Lalouette L., Pottier M.A., Wycke M.A., Boitard C., Bozzolan F., Maria A., Demondion E., Chertemps T., Lucas P., Renault D. (2016). Unexpected effects of sublethal doses of insecticide on the peripheral olfactory response and sexual behavior in a pest insect. Environ. Sci Pollut. Res. Int..

[6] de França S.M., Breda M.O., Barbosa D.R.S., Araujo A.M.N., Guedes C.A., Shields V.D.S. (2017). The Sublethal Effects of Insecticides in Insects. Biological Control of Pest and Vector Insects.

[7] Müller C. (2018). Impacts of sublethal insecticide exposure on insects—Facts and knowledge gaps. Basic Appl. Ecol..

[8] Xu C., Zhang Z., Cui K., Zhao Y., Han J., Liu F., Mu W. (2016). Effects of Sublethal Concentrations of Cyantraniliprole on the Development, Fecundity and Nutritional Physiology of the Black Cutworm *Agrotis ipsilon* (Lepidoptera: Noctuidae). PLoS ONE.

[9] Li J.-Y., Chen Y.-T., Wang Q.-Y., Zheng L.-Z., Fu J.-W., Shi M.-Z. (2022). Sublethal and Transgenerational Toxicities of Chlorfenapyr on Biological Traits and Enzyme Activities of *Paracoccus marginatus* (Hemiptera:Pseudococcidae). Insects.

[10] Bass C., Jones M. (2018). Editorial overview: Pests and resistance: Resistance to pesticides in arthropod crop pests and disease vectors: Mechanisms, models and tools. Curr. Opin. Insect. Sci..

[11] Simon-Delso N., Amaral-Rogers V., Belzunces L.P., Bonmatin J.M., Chagnon M., Downs C., Furlan L., Gibbons D.W., Giorio C., Girolami V. (2015). Systemic insecticides (neonicotinoids and fipronil): Trends, uses, mode of action and metabolites. Environ. Sci. Pollut. Res. Int..

[12] Sparks T.C., Crossthwaite A.J., Nauen R., Banba S., Cordova D., Earley F., Ebbinghaus-Kintscher U., Fujioka S., Hirao A., Karmon D. (2020). Insecticides, biologics and nematicides: Updates to IRAC’s mode of action classification—A tool for resistance management. Pestic. Biochem. Physiol..

[13] Eremina O.Y. (2017). Chlorfenapyr—Perspective pyrrole insecticide for combating resistant synanthropic insects. Pest. Manag..

[14] Chien S.-C., Chien S.-C., Su Y.-J. (2022). A fatal case of chlorfenapyr poisoning and a review of the literature. J. Int. Med. Res..

[15] Levchenko M.A., Silivanova E.A. (2019). Synergistic and antagonistic effects of insecticide binary mixtures against house flies (*Musca domestica*). Regul. Mech. Biosyst..

[16] Li X., Schuler M.A., Berenbaum M.R. (2007). Molecular mechanisms of metabolic resistance to synthetic and natural xenobiotics. Annu. Rev. Entomol..

[17] Zhang Y., Guo M., Ma Z., You C., Gao X., Shi X. (2020). Esterase-mediated spinosad resistance in house flies *Musca domestica* (Diptera: Muscidae). Ecotoxicology.

[18] Gong Y., Li M., Li T., Liu N. (2022). Molecular and functional characterization of three novel carboxylesterases in the detoxification of permethrin in the mosquito, *Culex quinquefasciatus*. Insect Sci..

[19] Serebrov V.V., Bakhvalov S.A., Glupov V.V. (2005). Induction of esterases in larvae of gypsy moth (*Lymantria dispar* L.) during infection by fungus metarhizium anisopliae (Metsch.) SOR. Euroasian Entomol. J..

[20] Yan S., Cui F., Qiao C. (2009). Structure, function and applications of carboxylesterases from insects for insecticide resistance. Protein Pept. Lett..

[21] Feng X., Liu N. (2020). Functional Analyses of House Fly Carboxylesterases Involved in Insecticide Resistance. Front. Physiol..

[22] Grigoraki L., Balabanidou V., Meristoudis C., Miridakis A., Ranson H., Swevers L., Vontas J. (2016). Functional and immunohistochemical characterization of CCEae3a, a carboxylesterase associated with temephos resistance in the major arbovirus vectors *Aedes aegypti* and *Ae*. Albopictus. Insect Biochem. Mol. Biol..

[23] Li Y., Liu J., Lu M., Ma Z., Cai C., Wang Y., Zhang X. (2016). Bacterial Expression and Kinetic Analysis of Carboxylesterase 001D from *Helicoverpa armigera*. Int. J. Mol. Sci..

[24] Walsh S.B., Dolden T.A., Moores G.D., Kristensen M., Lewis T., Devonshire A.L., Williamson M.S. (2001). Identification and characterization of mutations in housefly (*Musca domestica*) acetylcholinesterase involved in insecticide resistance. Biochem. J..

[25] Feyereisen R., Dermauw W., Van Leeuwen T. (2015). Genotype to phenotype, the molecular and physiological dimensions of resistance in arthropods. Pestic. Biochem. Physiol..

[26] Kim Y.H., Lee S.H. (2018). Invertebrate acetylcholinesterases: Insights into their evolution and non-classical functions. J. Asia-Pac. Entomol..

[27] Freitas A.P., Santos C.R., Sarcinelli P.N., Silva Filho M.V., Hauser-Davis R.A., Lopes R.M. (2016). Evaluation of a Brain Acetylcholinesterase Extraction Method and Kinetic Constants after Methyl-Paraoxon Inhibition in Three Brazilian Fish Species. PLoS ONE.

[28] Khan H.A., Akram W., Iqbal J., Naeem-Ullah U. (2015). Thiamethoxam Resistance in the House Fly, *Musca domestica* L.: Current Status, Resistance Selection, Cross-Resistance Potential and Possible Biochemical Mechanisms. PLoS ONE.

[29] Chang K.S., Kim H.C., Klein T.A., Ju Y.R. (2017). Insecticide resistance and cytochrome-P450 activation in unfed and blood-fed la-boratory and field populations of *Culex pipiens pallens*. J. Pest. Sci..

[30] Zhang Y., Wang Y., Ma Z., Zhai D., Gao X., Shi X. (2019). Cytochrome P450 monooxygenases-mediated sex-differential spinosad resistance in house flies *Musca domestica* (Diptera: Muscidae). Pest. Biochem. Physiol..

[31] Low V.L., Chen C.D., Lee H.L., Tan T.K., Chen C.F., Leong C.S., Lim Y.A.L., Lim P.E., Norma-Rashid Y., Sofian-Azirun M. (2013). Enzymatic Characterization of Insecticide Resistance Mechanisms in Field Populations of Malaysian *Culex quinquefasciatus* Say (Diptera: Culicidae). PLoS ONE.

[32] Amelia-Yap Z.H., Sofian-Azirun M., Chen C.D., Suana I.W., Lau K.W., Elia-Amira N.M.R., Haziqah-Rashid A., Tan T.K., Lim Y.A.L., Low V.L. (2019). Pyrethroids Use: Threats on Metabolic-Mediated Resistance Mechanisms in the Primary Dengue Vector *Aedes aegypti* (Diptera: Culicidae). J. Med. Entomol..

[33] Aponte A., Penilla R.P., Rodríguez A.D., Ocampo C.B. (2019). Mechanisms of pyrethroid resistance in *Aedes (Stegomyia) aegypti* from Colombia. Acta Trop..

[34] Scott J.G., Warren W.C., Beukeboom L.W., Bopp D., Clark A.G., Giers S.D., Hediger M., Jones A.K., Kasai S., Leichter C.A. (2014). Genome of the house fly, *Musca domestica* L., a global vector of diseases with adaptations to a septic environment. Genome Biol..

[35] Freeman J.C., Ross D.H., Scott J.G. (2019). Insecticide resistance monitoring of house fly populations from the United States. Pestic. Biochem. Physiol..

[36] Alam M., Shah R.M., Shad S.A., Binyameen M. (2020). Fitness cost, realized heritability and stability of resistance to spiromesifen in house fly, *Musca domestica* L. (Diptera: Muscidae). Pestic. Biochem. Physiol..

[37] Silivanova E.A., Levchenko M.A., Bikinyaeva R.K., Gavrichkin A.A. (2019). Efficacy of Chlorfenapyr against *Musca domestica* (Diptera: Muscidae): A Laboratory Study. J. Entomol. Sci..

[38] Shumilova P.A., Sennikova N.A., Silivanova E.A., Levchenko M.A. (2021). Biological responses in *Musca domestica* to fipronil and chlorfenapyr exposures. Regul. Mech. Biosyst..

[39] Ministry of Health of Brazil (2006). Quantification Methodology for Enzyme Activity Related to Insecticide Resistance in Aedes aegypti.

[40] Glavan G., Kos M., Božič J., Drobne D., Sabotič J., Kokalj A.J. (2018). Different response of acetylcholinesterases in salt- and detergent-soluble fractions of honeybee haemolymph, head and thorax after exposure to diazinon. Comp. Biochem. Physiol. C Toxicol. Pharmacol..

[41] Brown A.M. (2001). A step-by-step guide to non-linear regression analysis of experimental data using a Microsoft Excel spreadsheet. Comput. Methods Programs Biomed..

[42] Marasovic M., Marasovic T., Milos M. (2017). Robust Nonlinear Regression in Enzyme Kinetic Parameters Estimation. J. Chem..

[43] World Health Organization (2022). Manual for Monitoring Insecticide Resistance in Mosquito Vectors and Selecting Appropriate Interventions.

[44] Carvalho S.M., Belzunces L.P., Carvalho G.A., Brunet J.L., Badiou-Beneteau A. (2013). Enzymatic biomarkers as tools to assess environmental quality: A case study of exposure of the honeybee *Apis mellifera* to insecticides. Environ. Toxicol. Chem..

[45] Rumpf S., Hetzel F., Frampton C. (1997). Lacewings (Neuroptera: Hemerobiidae and Chrysopidae) and Integrated Pest Management: Enzyme Activity as Biomarker of Sublethal Insecticide Exposure. J. Econom. Entomol..

[46] Siddiqui J.A., Luo Y., Sheikh U.A.A., Bamisile B.S., Khan M.M., Imran M., Hafeez M., Ghani M.I., Lei N., Xu Y. (2022). Transcriptome analysis reveals differential effects of beta-cypermethrin and fipronil insecticides on detoxification mechanisms in *Solenopsis invicta*. Front. Physiol..

[47] Shi M.A., Yuan J.Z., Wu J., Zhuang P.J., Wu X.F., Tang Z.H. (2002). Kinetic Analysis of Acetylcholinesterase in a Propoxur-Resistant Strain of Housefly (*Musca domestica*) from Shanghai, China. Pestic. Biochem. Physiol..

[48] Zhang L., Shi J., Shi X., Liang P., Gao J., Gao X. (2010). Quantitative and qualitative changes of the carboxylesterase associated with beta-cypermethrin resistance in the housefly, *Musca domestica* (Diptera: Muscidae). Comp. Biochem. Physiol. B Biochem. Mol. Biol..

[49] Li Q., Huang J., Yuan J. (2018). Status and preliminary mechanism of resistance to insecticides in a field strain of housefly (*Musca domestica*, L.). Rev. Bras. Entomol..

[50] Riaz B., Kashif Z.M., Malik K., Ahmad A., Majeed H.N., Jabeen F., Zulhussnain M., Ranian K. (2022). Frequency of Pyrethroid Insecticide Resistance kdr Gene and Its Associated Enzyme Modulation in Housefly, *Musca domestica* L. Populations From Jhang, Pakistan. Front. Environ. Sci..

[51] Ma Z., Li J., Zhang Y., Shan C., Gao X. (2017). Inheritance mode and mechanisms of resistance to imidacloprid in the house fly *Musca domestica* (Diptera:Muscidae) from China. PLoS ONE.

[52] Farooq M., Freed S. (2018). Mortality, Biological, and Biochemical Response of *Musca domestica* (Diptera: Muscidae) to Selected Insecticides. J. Entomol. Sci..

[53] Zhao Y., Wang Q., Ding J., Wang Y., Zhang Z., Liu F., Mu W. (2018). Sublethal effects of chlorfenapyr on the life table parameters, nutritional physiology and enzymatic properties of *Bradysia odoriphaga* (Diptera: Sciaridae). Pestic. Biochem. Physiol..

[54] Xiao C., Luan S., Xu Z., Lang J., Rao W., Huang Q. (2017). Tolerance potential of *Chilo suppressalis* larvae to fipronil exposure via the modulation of detoxification and GABA responses. J. Asia-Pac. Entomol..

[55] Dewer Y., Pottier M.A., Lalouette L., Maria A., Dacher M., Belzunces L.P., Kairo G., Renault D., Maibeche M., Siaussat D. (2016). Behavioral and metabolic effects of sublethal doses of two insecticides, chlorpyrifos and methomyl, in the Egyptian cotton leafworm, *Spodoptera littoralis* (Boisduval) (Lepidoptera: Noctuidae). Environ. Sci. Pollut. Res. Int..

[56] Roat T.C., Carvalho S.M., Palma M.S., Malaspina O. (2017). Biochemical response of the Africanized honeybee exposed to fipronil. Environ. Toxicol. Chem..

[57] Feng X., Li M., Liu N. (2018). Carboxylesterase genes in pyrethroid resistant house flies, *Musca domestica*. Insect Biochem. Mol. Biol..

[58] You C., Shan C., Xin J., Li J., Ma Z., Zhang Y., Zeng X., Gao X. (2020). Propoxur resistance associated with insensitivity of acetylcholinesterase (AChE) in the housefly, *Musca domestica* (Diptera: Muscidae). Sci. Rep..

[59] Abobakr Y., Al-Hussein F.I., Bayoumi A.E., Alzabib A.A., Al-Sarar A.S. (2022). Organophosphate Insecticides Resistance in Field Populations of House Flies, *Musca domestica* L.: Levels of Resistance and Acetylcholinesterase Activity. Insects.

[60] Margus A., Piiroinen S., Lehmann P., Grapputo A., Gilbert L., Chen Y.H., Lindström L. (2021). Sequence variation and regulatory variation in acetylcholinesterase genes contribute to insecticide resistance in different populations of *Leptinotarsa decemlineata*. Ecol. Evol..

[61] Vang J.Y., Breceda C., Her C., Krishnan V.V. (2022). Enzyme kinetics by real-time quantitative NMR (qNMR) spectroscopy with progress curve analysis. Anal Biochem..

